# Loneliness and Problematic Social Media Use Among Adolescents: The Mediating Role of Fear of Missing Out

**DOI:** 10.3390/brainsci16070724

**Published:** 2026-07-08

**Authors:** Marianna Chmiel, Marek Cieśluk, Jan Znajdek

**Affiliations:** Institute of Psychology, University of Szczecin, 71-017 Szczecin, Poland

**Keywords:** problematic social media use, loneliness, fear of missing out, adolescents

## Abstract

**Background/Objectives**: Social media use is central to adolescent functioning, yet problematic engagement is linked to adverse psychosocial outcomes. This study examined the relationship between loneliness and problematic social media use (PSMU), investigating the role of fear of missing out (FoMO) as an underlying mechanism. **Methods**: The study included 206 Polish adolescents (*M* = 17.21, *SD* = 1.16). Participants completed the Bergen Social Media Addiction Scale, the Fear of Missing Out Scale, and the de Jong Gierveld Loneliness Scale. Correlation, multiple regression, and bootstrapping analyses were performed to test these associations. **Results**: Loneliness was positively associated with PSMU and specific FoMO dimensions. PSMU correlated with all FoMO dimensions, although the association with fear of social exclusion was weaker. Social comparison anxiety and online monitoring were identified as key factors associated with the indirect path from loneliness to PSMU (*β* = 0.09, 95% CI [0.03, 0.23] and *β* = 0.05, 95% CI [0.01, 0.15], respectively). Fear of social exclusion did not show a significant association with loneliness. **Conclusions**: These findings align with a compensatory framework, suggesting that loneliness is related to PSMU through specific cognitive and behavioral processes related to FoMO. Given the cross-sectional design, these results are exploratory; future longitudinal research is required to establish the directionality and temporal stability of these associations.

## 1. Introduction

Adolescence is a transitional stage that marks the end of the carefree period of childhood and prepares the individual for the challenges of adulthood. This period typically spans from approximately 12 to 20 years of age and is commonly divided into early and late phases, with late adolescence conventionally beginning around the age of 16 [1]. During this stage, changes in cognitive and personality domains enable adolescents to perceive themselves and their surrounding reality in a more complex and accurate manner [2].

The developmental tasks faced by adolescents include the formation and adoption of gender roles, acceptance of one’s physical appearance and body, internalization of values, increasing independence from parents and other adults, the ability to establish more mature peer relationships and preparation for a professional career [3]. These tasks are intended to facilitate a satisfying life based on goal setting and goal pursuit, effective coping with difficulties, and a sense of fulfillment derived from stable and intimate interpersonal relationships. Nowadays, the digital environment also constitutes an important context within which young people explore these developmental tasks.

The digital environment (including social media, advertising and virtual content) may constitute a potential risk factor in the process of identity consolidation, influencing adolescents’ patterns of thinking and emotional experiences [4,5]. Researchers suggest that because identity formation and other psychological changes are still underway during adolescence, young people may be particularly sensitive to the negative consequences of social media use [5], as well as the maturation of neural structures and circuits associated with emotion regulation and motivation [6]. Difficulty disengaging from social media may, in turn, affect the formation of social relationships and lead to negative emotional consequences [7].

The expansion of digital technologies has made internet access and social media platforms an increasingly common part of adolescents’ everyday lives, contributing to greater engagement in online activities [8]. Social media platforms (e.g., Instagram, Snapchat, or TikTok) have become key tools for adolescents’ communication, relationship building, and perceptions of themselves and the world [9]. At the same time, social media may also have positive functions for young people, such as fostering a sense of belonging, facilitating access to social support, enhancing communication skills and enabling self-expression [5,10,11,12]. Nevertheless, researchers emphasize that excessive and/or problematic use of the internet and social media may lead to adverse effects on health and well-being and may be associated with the risk of developing addictive behaviors [13,14]. In the literature and previous research, it is therefore possible to identify a still-existing research gap concerning psychological factors associated with problematic social media use among adolescents.

### 1.1. Problematic Social Media Use

With adolescents using social media more and more intensively, the term ‘problematic social media use’ has become widely discussed in both scientific and mainstream literature. It is worth noting that neither the ICD-11 nor the DSM-5 includes diagnostic categories such as “social media addiction” or “internet addiction,” nor do they recognize “harmful use of social media” as a formal disorder [15,16]. The lack of definitional consensus and variability in operationalization across studies complicate the establishment of uniform criteria and diagnosis [14]. An important theoretical approach was proposed by Davis, who distinguished between generalized and specific forms of problematic internet use [17].

Furthermore, the literature indicates that overly general measures (e.g., total time spent online) may yield less consistent findings in relation to negative psychophysical states than measures referring to specific online activities. For instance, evidence from research involving adolescents aged 14 years in the United Kingdom indicated an association between depressive symptoms and social media use, while no such relationship was observed with overall time spent online [18]. Excessive social media use is also frequently interpreted within the framework of Griffiths’ components model, which describes problematic behaviors in terms of six addiction-related components: salience, mood modification, tolerance, withdrawal, conflict, and relapse [19].

In Poland, maladaptive patterns of internet and social networking platform use affect a significant proportion of adolescents [8]. Research indicates that problematic social media use is associated, among other factors, with poorer mental functioning and well-being [14,20], symptoms of addictive use [21,22], mood disturbances and depression [18,23], lower resilience [24], and fear of missing out (FoMO) [25]. In light of recent research findings, it can be hypothesized that certain psychological factors may either directly interact with problematic social media use or contribute to this association.

### 1.2. The Compensatory Mechanism of Social Media Use

To understand the mechanisms underlying problematic social media use, it is crucial to examine the psychological factors that drive individuals toward maladaptive online behaviors. According to Self-Determination Theory (SDT) [26], individuals who experience deficits in their basic psychological needs, namely autonomy, competence, and relatedness, actively seek alternative pathways to satisfy them. Drawing upon the Compensatory Internet Use Model [27], it can be assumed that psychological deficiencies experienced in the real world will be compensated for in the virtual environment to regulate one’s mood. Within the scope of this study, the focus is placed on the deficit of the need for relatedness, assuming that it manifests through a sense of loneliness. It is posited that loneliness, along with the phenomenon of FoMO, interacts with problematic social media use.

In this study, loneliness is conceptualized in accordance with the framework proposed by de Jong-Gierveld [28]. Loneliness is understood as a subjective experience resulting from a discrepancy between an individual’s actual and desired social relationships, both in quantitative and qualitative terms. Following the de Jong Gierveld framework, we conceptualize loneliness as a multidimensional construct encompassing both social and emotional deficits, which extends beyond the simple dichotomy of physical vs. virtual isolation. This conceptualization distinguishes three key dimensions of loneliness: (1) the type of deprivation (i.e., the nature and intensity of missing relationships), (2) the temporal perspective (transient versus chronic), and (3) the emotional characteristics (the co-occurrence of negative affect in the absence of positive affect).

The existing literature does not provide unequivocal evidence regarding the connection between the intensity of social media use and loneliness. Some studies indicate a positive association between the intensity of use of specific social media platforms and increased intensity of subjective loneliness [29,30]. However, this relationship is not universal and does not apply equally across all social networking services. Divergent findings emerge from studies focusing on specific applications, such as Instagram, where certain types of user activity have been linked to reduced levels of loneliness [31].

These inconsistencies suggest that, in the context of psychological well-being, it is not merely the use of digital technologies per se that is of primary importance, but rather the specific nature and form of engagement, as well as individual user characteristics.

FoMO is commonly understood as a persistent unease stemming from the belief that others are enjoying gratifying experiences from which an individual feels excluded. Furthermore, we approach FoMO as a multifaceted psychological state that functions as a bridge between social needs and digital engagement, rather than a phenomenon limited to online-only relational dynamics. This construct is characterized not only by an emotional component but also by a behavioral tendency, namely the desire to remain continuously connected with what others are doing. According to the original conceptualization [32], FoMO is closely related to SDT. It may be interpreted as a state of “self-regulatory limbo,” resulting from temporary or a long-term failure to satisfy basic psychological needs.

Insufficient need satisfaction may drive social media engagement through two pathways: directly, as social media enable contact with others, support the cultivation of social skills, and facilitate the enhancement of social bonds, and indirectly, through the mechanism of FoMO. Within this framework, FoMO is conceptualized as a mediator linking unmet psychological needs with engagement in social media [25].

These assumptions are supported by a growing body of empirical evidence. FoMO has been shown to have stronger predictive power than traditional indicators of psychopathology, such as anxiety and depression, in explaining problematic smartphone use [32]. Moreover, FoMO is positively associated with problematic internet and Instagram use among adolescents [33,34]. It has also been demonstrated that FoMO mediates the relationship between anxiety and depression, and the negative consequences of mobile device use in adolescent populations [35]. Additionally, research indicates that loneliness is positively associated with FoMO [36].

Taken together, these findings highlight the underlying role of FoMO as a mediator connecting loneliness and problematic social media use (PSMU), which warrants further empirical investigation.

**Figure 1:**
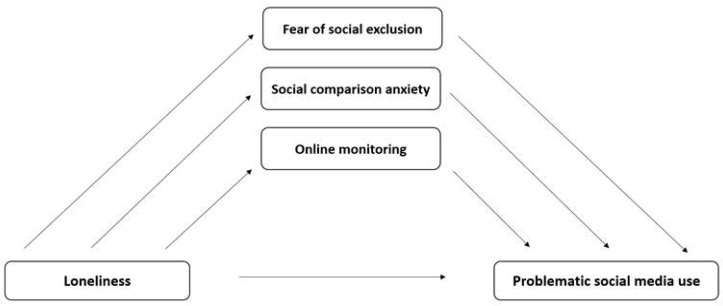
A conceptual framework of indirect effects involving fear of missing out in the associations between loneliness and problematic social media use.

The primary aim of this study was to empirically test the compensatory hypothesis of social media use by examining a conceptual model of the associations between loneliness, fear of missing out (FoMO), and problematic social media use (PSMU) among adolescents (Figure 1). Specifically, the study examined whether loneliness is positively associated with problematic social media use (H1) and fear of missing out (H2), and whether the dimensions of fear of missing out are positively related to problematic social media use (H3). Finally, it was hypothesized that the dimensions of fear of missing out might serve as potential indirect pathways linking loneliness and problematic social media use (H4). Due to the cross-sectional design of this study, establishing causal relationships is not possible; consequently, our examination of indirect effects should be viewed as exploratory and grounded in existing theoretical frameworks.

## 2. Materials and Methods

### 2.1. Participants

The study involved 206 Polish secondary school students, aged between 15 and 19 years (*M* = 17.21; *SD* = 1.16). Regarding academic progression, 56.8% of the participants were in the fourth grade, while 25.2% and 18.0% were in the second and third grades, respectively. The gender distribution was 61.2% female and 38.8% male. The geographic diversity of the sample was notable: 40.8% of respondents lived in cities with 150,000–400,000 inhabitants, 24.8% in rural areas, 18.9% in cities exceeding 400,000, 13.1% in towns with up to 50,000 residents, and 2.4% in cities with between 50,000 and 150,000 inhabitants. Regarding family structure, the majority of participants (78.6%) came from two-parent families, 16.5% from single-parent families, and 4.9% from reconstructed families. As for current living arrangements, most adolescents (82.5%) lived in the family home with parents or guardians, 13.6% resided in a school dormitory, 2.9% lived with siblings or other adults, and a small proportion lived in rented accommodation or other arrangements (1%).

To provide additional context regarding participants’ social media use, descriptive data on preferred platforms and usage patterns were collected. The majority of adolescents reported spending the most time on TikTok (46.1%), followed by Instagram (26.7%) and YouTube (17.5%). Other platforms, including Facebook, Twitter and communication applications such as Messenger, were reported less frequently and were grouped as less commonly used platforms. Regarding the time of day of social media use, most participants indicated that they used social media primarily in the evening (35.9%) or in the afternoon, directly after school (31.6%). A notable proportion also reported frequent use late in the evening before going to sleep (18.0%). Other patterns of use (e.g., morning, during classes, or evenly throughout the day) were relatively infrequent and were therefore treated as marginal categories. In addition to categorical indicators, several quantitative measures of media use were assessed. On average, participants reported spending 3.76 h per day on social media (*SD* = 1.77). Time spent on other screen-based activities (e.g., gaming, television, other applications) amounted to an average of 3.21 h daily (*SD* = 2.80). The mean duration of social media use experience was 6.83 years (*SD* = 2.43), indicating that most adolescents had been exposed to social media since late childhood. Furthermore, participants reported a moderate tendency to use social media while engaging in other activities (e.g., studying or completing household tasks), with a mean score of 2.92 (*SD* = 1.00) on a five-point scale.

Additionally, participants were asked to indicate which social media platforms they use regularly (multiple responses were allowed). The most commonly used platforms were Instagram (91.3%), YouTube (81.6%), and TikTok (72.8%). Less frequently reported platforms included Facebook (42.7%), Snapchat (26.7%), and Twitter (17.0%), while other platforms were indicated only sporadically and were grouped as marginal categories. Participants were also asked about their main purposes for using social media (up to five responses could be selected). The most frequently reported motives were entertainment and scrolling (90.3%) and maintaining contact with friends and family (77.7%). A substantial proportion of adolescents also reported using social media for educational or informational purposes (60.7%) and for following news (50.0%). Additionally, 40.8% indicated seeking inspiration or developing hobbies, and 40.3% reported using social media as a way of passing time. Other motives, such as content creation, making purchases, or forming new relationships, were less commonly endorsed.

### 2.2. Procedure

Data collection occurred in a Polish high school during March 2026, where students completed an online survey during scheduled class hours. To ensure ethical compliance, informed consent was mandatory for all participants; parental authorization was required for underage students and secured via homeroom teachers, whereas adult participants provided their own consent. The survey employed established psychometric scales to measure the study variables. Participation was strictly voluntary, permitting students to withdraw at their discretion. All individual responses were handled under conditions of anonymity and strict confidentiality. The data acquisition phase concluded within a one-month period, and no financial remuneration was provided for involvement. The project approval for the current study was obtained from The University Research Ethics Committee of the Institute of Psychology at the University of Szczecin (No. 1/2026, 22 January 2026) and was conducted according to the standards of the Declaration of Helsinki.

### 2.3. Measures

#### 2.3.1. Bergen Social Media Addiction Scale

The Bergen Social Media Addiction Scale (BSMAS) is a self-report instrument designed to assess social media addiction, conceptualized as a behavioral addiction characterized by salience, mood modification, tolerance, withdrawal, conflict, and relapse, based on Griffiths’ model [19]. It was developed by Andreassen and colleagues [37] and adapted for Polish conditions by Balcerowska and colleagues [38]. It consists of six statements related to social media engagement (e.g., “How often during the last year have you used social networking sites in order to forget about personal problems?”). Participants rate each statement on a five-point Likert scale ranging from 1 (very rarely) to 5 (very often). Higher total scores indicate stronger engagement in behaviors consistent with social media addiction. In the present study, the term problematic social media use (PSMU) is used instead of “social media addiction” to emphasize problematic patterns of engagement without implying a clinical diagnosis. The reliability coefficient for the present study was Cronbach’s alpha = 0.721.

#### 2.3.2. Fear of Missing Out (FoMO) Scale

The Fear of Missing Out (FoMO) scale is a self-report instrument originally developed by Przybylski and colleagues [25] to measure the intensity of fear of missing out, and subsequently adapted into Polish by Rodzeń [39] as part of an unpublished master’s thesis at the University of Szczecin. The scale consists of ten statements grouped into three subscales: (1) Fear of social exclusion (e.g., “It is important that I understand my friends ‘in jokes’”), (2) Social comparison anxiety (e.g., “I fear my friends have more rewarding experiences than me”) and (3) Online monitoring (e.g., “Sometimes, I wonder if I spend too much time keeping up with what is going on”). Participants rate each statement on a five-point Likert scale ranging from 1 (Not at all true of me) to 5 (Extremely true of me). Higher total scores indicate greater intensity of fear of missing out. The reliability coefficients for the present study were Cronbach’s alpha = 0.787 for the total scale, Cronbach’s alpha = 0.564 for Fear of social exclusion, Cronbach’s alpha = 0.835 for Social comparison anxiety, and Cronbach’s alpha = 0.600 for Online monitoring.

#### 2.3.3. De Jong Gierveld Loneliness Scale

The de Jong Gierveld Loneliness Scale (DJGLS) is a self-report instrument designed to measure perceived loneliness. It was developed by de Jong Gierveld and van Tilburg [40] and adapted for Polish conditions by Grygiel and colleagues [41]. The scale consists of eleven statements rated on a five-point Likert scale from 1 (none of the time) to 5 (all of the time). It includes two subscales measuring emotional loneliness (e.g., “I miss having a really close friend”) and social loneliness (e.g., “There are plenty of people I can lean on when I have problems,” reverse-scored); however, in the present study the overall total score was used without distinguishing between subscales. Higher total scores indicate higher levels of loneliness. The reliability coefficient for the present study was Cronbach’s alpha = 0.857.

### 2.4. Data Analysis

Prior to the main analyses, an a priori power calculation was performed using G*Power 3.1.9.7 [42]. A linear multiple regression model (fixed model, $R^{2}$ increase) was specified to determine the required sample size. To achieve a power (1 − *β*) of 0.95 and detect a medium effect size ($f^{2}$ = 0.15) at a significance level of *α* = 0.05, the analysis indicated a minimum requirement of 119 participants. A final sample of 206 individuals was collected to increase representativeness regarding the population of Polish adolescents and to enhance the precision of effect size estimations.

Regression diagnostics were executed to evaluate the influence of individual observations, specifically by examining Cook’s distance, Mahalanobis distance, and leverage (centered influence value). While a small number of cases exhibited relatively high Mahalanobis distance and leverage values, their Cook’s distance values remained consistently low. This indicates that no single observation exerted undue influence on the model estimates. Consequently, the regression model appears robust, with no evidence of distortion attributable to outliers.

To verify the normality of the distributions for the study variables, specifically problematic social media use (PSMU), loneliness, and the dimensions of fear of missing out (fear of social exclusion, social comparison anxiety, and online monitoring), skewness and kurtosis coefficients were examined. Since all values remained within the acceptable ±1 range it was concluded that the data did not deviate significantly from a normal distribution. Consequently all continuous variables were retained in their original format, applying no categorization or transformations to the quantitative data. Furthermore, multicollinearity among predictors was inspected by calculating tolerance values and variance inflation factors (VIFs). Tolerance scores ranged between 0.602 and 0.806, while VIF values fell between 1.24 and 1.66. These indices reside well within conventional thresholds, confirming that multicollinearity was not a confounding factor in the analyses.

A hierarchical regression analysis was conducted to examine whether selected sociodemographic and social media use variables (sex, age, class, place of residence, family structure, living arrangements, daily social media use, duration of social media use, time of day of social media use, multitasking during social media use and other screen-based activities) acted as potential confounders in explaining problematic social media use. In the first step, these variables jointly accounted for approximately 37.5% of the variance (*R*^2^ = 0.375; adjusted *R*^2^ = 0.336), with sex (*β* = −0.269, *p* < 0.001), daily social media use (*β* = 0.183, *p* = 0.004), time of day of social media use (*β* = 0.118, *p* = 0.044), and multitasking during social media use (*β* = 0.386, *p* < 0.001) reaching statistical significance. In the second step, psychological variables were included: loneliness, fear of social exclusion, social comparison anxiety, and online monitoring.

The explained variance increased substantially (*R*^2^ = 0.487; adjusted *R*^2^ = 0.444). After adding these variables, some of the sociodemographic and behavioral predictors lost statistical significance, whereas loneliness (*β* = 0.162, *p* = 0.007), social comparison anxiety (*β* = 0.162, *p* = 0.022), online monitoring (*β* = 0.214, *p* = 0.003), daily social media use (*β* = 0.174, *p* = 0.003), and multitasking during social media use (*β* = 0.272, *p* < 0.001) remained significant predictors. These results suggest that psychological factors contribute independently to problematic social media use, beyond the effects of sociodemographic and behavioral characteristics.

Data were analyzed using IBM SPSS Statistics, version 28. Initially, two-tailed correlations were computed to identify preliminary associations between the study variables. Subsequently, to investigate whether fear of social exclusion, social comparison anxiety, and online monitoring contributed to the indirect association between loneliness and problematic social media use, indirect effect analyses were performed. Consistent with the guidelines provided by Hayes [43], these models were tested using Model 4, applying bootstrapping with 5000 iterations and 95% bias-corrected confidence intervals.

## 3. Results

In line with our theoretical framework, the following results examine the associations between loneliness, FoMO dimensions, and problematic social media use, testing the hypothesis that FoMO-related processes may act as mechanisms of compensatory social media engagement.

### 3.1. Initial Correlations Among Variables

To begin the analysis, Pearson correlation coefficients were calculated to assess the associations between problematic social media use, fear of missing out, fear of social exclusion, social comparison anxiety, online monitoring, and loneliness. Additionally, descriptive statistics, including means and standard deviations, were derived for all variables. These results are summarized in Table 1.

Most variables were significantly correlated. Problematic social media use showed positive associations with fear of missing out, social comparison anxiety, online monitoring and loneliness, with a weaker association observed for fear of social exclusion. Fear of missing out was strongly related to all subcomponents of FoMO, including fear of social exclusion, social comparison anxiety and online monitoring. Loneliness was positively associated with problematic social media use, fear of missing out, social comparison anxiety and online monitoring, and its association with fear of social exclusion was not significant. The strength of these relationships ranged from weak to strong.

### 3.2. Analysis of Direct and Indirect Effects

To investigate the role of fear of missing out dimensions in the relationship between loneliness and problematic social media use, an indirect effects model was tested. Loneliness was specified as the predictor and problematic social media use as the outcome variable, with the three dimensions of fear of missing out (fear of social exclusion, social comparison anxiety and online monitoring) included as potential contributors to the indirect associations. This analysis was implemented via bootstrapping procedures (Model 4, 5000 resamples) to generate 95% bias-corrected confidence intervals [43]. The cross-sectional design of this study precludes the drawing of causal inferences. Consequently, these findings are best regarded as theory-driven, exploratory assessments of potential indirect associations. Furthermore, when interpreting these results, we emphasize both statistical significance and the practical relevance of the observed effect sizes, as statistically significant results with modest coefficients warrant cautious interpretation regarding their real-world impact. The results are presented in Table 2.

The regression model examining the associations between loneliness, dimensions of fear of missing out, and problematic social media use was statistically significant, *F*(4, 201) = 19.45, *p* < 0.001, and explained approximately 28% of the variance in problematic social media use (*R*^2^ = 0.28), which represents a moderate level of explained variance in the context of behavioral social science. Standardized beta coefficients (*β*) are reported alongside unstandardized 95% confidence intervals to provide both effect size and estimation precision.

Loneliness was positively associated with social comparison anxiety (*β* = 0.43, 95% CI [0.29, 0.53]) and online monitoring (*β* = 0.15, 95% CI [0.01, 0.26]), but not with fear of social exclusion (*β* = 0.01, 95% CI [−0.10, 0.12]). Among the three FoMO dimensions, social comparison anxiety (*β* = 0.21, 95% CI [0.08, 0.52]) and online monitoring (*β* = 0.34, 95% CI [0.29, 0.74]) were significant positive predictors of problematic social media use, whereas fear of social exclusion did not show a significant effect (*β* = −0.06, 95% CI [−0.31, 0.12]). While these coefficients are statistically significant, it should be noted that some of the observed effects, particularly for online monitoring as a predictor of PSMU, are modest in magnitude, suggesting that these factors contribute to, but do not fully determine, problematic engagement.

As a result, the total indirect effect of loneliness on problematic social media use via the FoMO dimensions was statistically significant (*β* = 0.14, 95% CI [0.08, 0.33]). Specifically, significant indirect effects were observed through social comparison anxiety (*β* = 0.09, 95% CI [0.03, 0.23]) and online monitoring (*β* = 0.05, 95% CI [0.01, 0.15]), whereas the indirect effect via fear of social exclusion was not significant (*β* = −0.00, 95% CI [−0.02, 0.02]). Although these indirect associations are statistically significant, their modest effect sizes suggest that they represent only partial pathways in the complex relationship between loneliness and problematic social media use.

Thus, social comparison anxiety and online monitoring appear to contribute significantly to the indirect pathway between loneliness and problematic social media use, while fear of social exclusion does not appear to play a meaningful role in this association. The direct effect of loneliness on problematic social media use was statistically significant (*β* = 0.16, 95% CI [0.04, 0.40]), indicating a partial indirect effect. The total effect was also significant (*β* = 0.30, 95% CI [0.23, 0.59]).

Overall, these findings suggest that social comparison anxiety and online monitoring may serve as significant mediating mechanisms linking loneliness to problematic social media use among adolescents, though the practical significance of these indirect pathways should be viewed with caution given the modest effect sizes observed. This pattern is consistent with a partial indirect effect and should be interpreted with caution given the cross-sectional design of the study.

## 4. Discussion

The aim of the present study was to examine the relationships between loneliness, fear of missing out (FoMO), and problematic social media use, as well as to determine whether specific dimensions of FoMO contribute to the indirect association between loneliness and problematic social media use. The obtained results largely supported the proposed hypotheses.

### 4.1. Explaining Associations Between Problematic Social Media Use and Loneliness

This study confirmed that loneliness is positively associated with problematic social media use (H1). This outcome corresponds with previous empirical research among adolescents, which has demonstrated that higher levels of loneliness are associated with greater problematic online activity, including problematic social media use and other forms of excessive internet use [44,45,46].

A convergent pattern also emerges from longitudinal studies and review papers, which suggest that this relationship may be bidirectional: loneliness may foster compensatory engagement in online activities, while excessive and problematic media use may, in turn, exacerbate social isolation and feelings of loneliness [47,48]. Collectively, these findings support the interpretation that social media may function as a compensatory coping strategy for unmet offline social needs, although this strategy may be maladaptive and reinforce psychosocial difficulties over time.

Furthermore, findings reported by Wu and colleagues [49] suggest that higher levels of loneliness and more pronounced problematic social media use may be connected to a greater likelihood of further escalation of both difficulties over time.

However, because the present study used a cross-sectional design, the direction of these relationships cannot be determined, and the observed associations between loneliness and problematic social media use may also reflect the influence of other psychological, social, or contextual factors.

### 4.2. Role of Fear of Missing Out in Associations Between Loneliness and Problematic Social Media Use

The present findings support the hypothesis of a positive connection between loneliness and FOMO (H2). The results indicate that individuals experiencing loneliness in offline contexts tend to report a greater tendency to feel discomfort related to the belief that they are excluded from the gratifying social activities of their peers. Notably, loneliness was most strongly associated with the FoMO dimension related to social comparison anxiety. However, this association was not uniform across all dimensions of the construct. It was observed that loneliness was not directly related to the fear of social exclusion dimension.

The finding that the global FoMO score is significantly associated with loneliness is strongly supported by existing literature. Similar correlations between overall FoMO levels and loneliness have been reported among adolescents [50] as well as young adults [51]. Additionally, Mao et al. [52] found that loneliness is more strongly associated with FoMO as a trait than with FoMO as a state.

The lack of a significant association between loneliness and the fear of social exclusion subscale is particularly noteworthy. This result may be interpreted in light of Hobfoll’s Conservation of Resources (COR) theory [53]. According to this framework, stress (and consequently anxiety) arises from the actual or anticipated loss of valued resources. Importantly, anxiety related to the loss of a specific resource (e.g., group belonging) presupposes that the individual currently possesses that resource. Individuals experiencing higher levels of loneliness may already perceive social bonds as absent or lost resources; consequently, the anticipation of exclusion may not trigger strong anxiety responses, as it concerns a resource that is no longer available to them.

The results of the present study also support hypothesis H3, according to which FoMO dimensions are positively associated with problematic social media use (PSMU). The analysis revealed statistically significant positive associations between PSMU and both the global FoMO score and all subdimensions. The strongest associations were observed for social comparison anxiety and online monitoring, whereas the relationship with fear of social exclusion was somewhat weaker, although still significant. These findings suggest that as FoMO increases, so does the intensity of problematic engagement with social media platforms.

These results align with prior research identifying FoMO as a significant predictor of PSMU in both adolescent populations [54,55,56] and young adults [57]. The associations between all FoMO dimensions and problematic social media use suggest a meaningful interplay between these constructs, which may be interpreted bidirectionally. On one hand, individuals may use online activity as a strategy to reduce anxiety and uncertainty; on the other hand, continuous exposure to social media content may further intensify FoMO, thereby reinforcing maladaptive patterns of use. This cyclical relationship is supported by previous studies highlighting the bidirectional nature of the association between FoMO and problematic social media engagement [57].

Building on these findings, the final hypothesis (H4) proposed that FoMO dimensions may function as potential indirect pathways linking loneliness and problematic social media use. The findings yielded partial support for this hypothesis, given that social comparison anxiety and online monitoring emerged as significant contributors to the indirect pathway, whereas fear of social exclusion did not.

This finding aligns with prior studies carried out among adult populations. Li et al. [58] demonstrated that FoMO mediates the daily interaction between loneliness and problematic social media use. Similarly, Wang et al. [59] found that FoMO is a significant mediator in the relationship between loneliness and problematic use of social networking sites, suggesting that this mechanism may operate across different forms of social media-related internet use.

The lack of a mediating effect of fear of social exclusion in the relationship between loneliness and problematic social media use may be explained by the specific motivational processes underlying social media engagement. Drawing on the Compensatory Internet Use model [27], individuals experiencing psychosocial difficulties such as loneliness may use social media as a compensatory strategy aimed at fulfilling unmet social needs or alleviating negative affective states. In this context, FoMO dimensions related to active information seeking and social comparison may play a more central mediating role than concerns about exclusion, as they reflect more active forms of engagement with alternative social experiences.

From this perspective, social media may represent an attempted compensatory response to unmet social needs in real-life, face-to-face interactions. However, when such engagement is driven by FoMO, social comparison, and continuous online monitoring, it may not necessarily reduce loneliness and may instead reinforce maladaptive patterns of social media use. Overall, the findings suggest that FoMO represents a key psychological mechanism linking loneliness to problematic social media use, particularly through its cognitive and monitoring-related dimensions.

It should be emphasized that the study was correlational in nature; therefore, a definitive causal relationship cannot be established. Although our model assumed that loneliness can lead to problematic social media use both directly and through the mechanism of FOMO, it is worth considering whether this relationship could be reversed. An experimental study by Hunt et al. [60] indicated that over a three-week period, a group of students who limited their social media use to 30 min per day showed a significant decrease in loneliness and depression compared to the control group. In another randomized controlled trial, deactivating Facebook accounts four weeks before the midterm elections was associated with increased offline activity, including social gatherings with family and friends [61]. Although the latter was not a psychological study, it can be assumed that a greater frequency of face-to-face meetings with family and friends is linked to a reduction in loneliness. The considerations discussed earlier, along with the aforementioned studies, suggest that the relationship between social media and loneliness is likely bidirectional. Therefore, in reality, this mechanism can be expected to involve a dynamic feedback loop.

### 4.3. Limitations and Future Implications

While this study provides valuable insights into the relationships between loneliness, fear of missing out (FoMO), and problematic social media use (PSMU), several limitations should be acknowledged.

First, the cross-sectional design precludes conclusions about causality or temporal ordering. Although FoMO dimensions were identified as potential mediators, these effects should be interpreted as exploratory rather than confirmatory. Longitudinal research is needed to verify the directionality of the proposed associations among loneliness, FoMO, and PSMU.

Second, the exclusive use of self-report measures may introduce common method bias and social desirability effects. Future studies should incorporate behavioral or digital trace data to obtain more objective indicators of social media engagement, particularly in relation to monitoring behaviors.

Third, several sampling limitations must be noted. The study utilized a relatively small, homogeneous sample of Polish adolescents recruited from a single school, which limits the generalizability of the findings compared to larger epidemiological studies. Additionally, the potential cultural influences specific to the Polish context may restrict the broader applicability of the results. Furthermore, the notable gender imbalance (61.2% female) within this sample warrants caution when extrapolating results to more diverse populations. Future research should prioritize larger, more diverse, and representative samples across different cultural and demographic settings.

Fourth, although the instruments used are well-established, some subscales of the FoMO scale, particularly ‘Fear of Social Exclusion’ and ‘Online Monitoring,’ exhibited relatively low internal reliability in the current sample. Consequently, the findings related to these specific dimensions should be interpreted with caution, as measurement error may have influenced the magnitude of the observed associations.

Despite these limitations, the findings highlight the importance of both cognitive (e.g., social comparison processes) and behavioral (e.g., online monitoring) mechanisms in understanding problematic social media use. Future research should examine these processes using longitudinal or experience-sampling designs to capture within-person dynamics. From an applied perspective, interventions may benefit from targeting maladaptive cognitive patterns. Such interventions could focus on modifying maladaptive beliefs related to the need for constant presence in the online environment, strengthening emotion regulation skills, and reducing automatic, habitual checking of social media. At the same time, they should support the development of more constructive ways of coping in offline interactions and more conscious and controlled use of social media. Similar conclusions were reached by Bağatarhan and Siyez [62], who showed that a cognitive-behavioral prevention program for internet addiction may be effective among adolescents, particularly when combined with parent psychoeducation. Furthermore, future research should investigate whether this compensatory process remains a transient coping strategy or evolves into a chronic, maladaptive pattern of social media engagement over time.

## 5. Conclusions

The present study offers empirical evidence linking loneliness to problematic social media use (PSMU) among adolescents, both directly and indirectly through specific dimensions of the Fear of Missing Out (FoMO). By demonstrating that “social comparison anxiety” and “online monitoring” (but not “fear of social exclusion”) contribute to indirect pathways, our findings provide a more nuanced understanding of the relationship between loneliness and digital engagement. This nuanced perspective advances current knowledge by moving beyond global measures of FoMO and highlighting the distinct cognitive and behavioral pathways through which loneliness may drive problematic social media behavior.

These results are in agreement with the Compensatory Internet Use framework, supporting the interpretation that adolescents experiencing loneliness may turn to social media as a strategy to mitigate unmet social needs. The findings confirm that this compensatory engagement is not a uniform process but is instead facilitated by specific manifestations of FoMO. Although our results align with this theoretical framework, the cross-sectional nature of the data dictates that these findings remain exploratory. The observed associations do not imply that compensatory social media use definitively reinforces loneliness; rather, they suggest that these variables operate within a complex, potentially bidirectional system.

Ultimately, this study underscores the importance of considering the multidimensional nature of FoMO when examining adolescent digital well-being. By clarifying that specific cognitive and monitoring-related processes act as potential pathways between loneliness and PSMU, our work highlights critical targets for future longitudinal research and intervention programs aimed at fostering more conscious social media engagement.

## Figures and Tables

**Table 1:** Descriptive statistics and correlations among problematic social media use (PSMU), fear of missing out (FoMO) subcomponents, and loneliness in adolescents (N = 206).

Variables	*M*	*SD*	1.	2.	3.	4.	5.
1. Problematic social media use	16.10	4.57	-				
2. Fear of missing out	24.68	6.94	0.45 **	-			
3. Fear of social exclusion	9.09	2.74	0.15 *	0.71 **	-		
4. Social comparison anxiety	7.37	3.20	0.44 **	0.79 **	0.28 **	-	
5. Online monitoring	8.22	2.96	0.45 *	0.83 **	0.42 **	0.52 **	-
6. Loneliness	4.26	3.34	0.30 **	0.27 **	0.01	0.43 **	0.15 *

** *p* < 0.01; * *p* < 0.05.

**Table 2:** Direct and indirect associations between loneliness and problematic social media use with dimensions of fear of missing out as potential intervening variables (N = 206).

Pathways	*β*	95% CI		SE	*t*
		LL	UL		
Direct effects					
Loneliness ⭢ Fear of social exclusion	0.01	−0.10	0.12	0.06	0.20
Loneliness ⭢ Social comparison anxiety	0.43 ***	0.29	0.53	0.06	6.74
Loneliness ⭢ Online monitoring	0.15 **	0.01	0.26	0.06	2.19
Fear of social exclusion ⭢ PSMU	−0.06	−0.31	0.12	0.11	−0.85
Social comparison anxiety ⭢ PSMU	0.21 **	0.08	0.52	0.11	2.73
Online monitoring ⭢ PSMU	0.34 ***	0.29	0.74	0.11	4.51
Loneliness ⭢ PSMU	0.16 **	0.04	0.40	0.09	2.37
Indirect effects					
Indirect total effect	0.14	0.08	0.33	0.06	-
Loneliness ⭢ Fear of social exclusion ⭢ PSMU	−0.00	−0.02	0.02	0.01	-
Loneliness ⭢ Social comparison anxiety ⭢ PSMU	0.09	0.03	0.23	0.05	-
Loneliness ⭢ Online monitoring ⭢ PSMU	0.05	0.01	0.15	0.04	-
Total effect					
Loneliness ⭢ PSMU	0.30 ***	0.23	0.59	0.09	4.46

Note. Standardized coefficients (*β*) are reported. 95% CI = 95% confidence interval for the unstandardized coefficient; SE = standard error; *t* = *t*-value. *** *p* < 0.001; ** *p* < 0.05.

## Data Availability

The raw data supporting the conclusions of this article will be made available by the authors on request due to its sensitive ethical nature.
